# A Modern Superstition in Disease; the Germ Theory Reconsidered

**Published:** 1889-09

**Authors:** 


### A Modern Superstition in Disease; The Germ Theory Reconsidered. By Lewis Sanders. New York, 1889.

This pamphlet of sixteen pages is, as its name indicates, an attack upon the germ theory of disease. The author begins his essay with the inquiry: Are bacteria the *causa causans* of disease? And then proceeds to demolish "this startling theory, hysterically advanced and fervently urged," and complains that "it has held possession too long of the throne of truth, imposing upon public credulity like so many of its empirical congeners."

He asserts that "the law of disease must be a unit; just as the laws of gravitation, of music, of painting, of light, and of sound are units, it is but the complement of the law of health."

The rest of the essay contains a number of unpleasant questions for the adherents of the germ theory to answer.

